# Intramolecular tautomerization of the quercetin molecule due to the proton transfer: QM computational study

**DOI:** 10.1371/journal.pone.0224762

**Published:** 2019-11-21

**Authors:** Ol’ha O. Brovarets’, Dmytro M. Hovorun

**Affiliations:** 1 Department of Molecular and Quantum Biophysics, Institute of Molecular Biology and Genetics, National Academy of Sciences of Ukraine, Kyiv, Ukraine; 2 Department of Molecular Biotechnology and Bioinformatics, Institute of High Technologies, Taras Shevchenko National University of Kyiv, Kyiv, Ukraine; University of Calgary, CANADA

## Abstract

Quercetin molecule (3, 3′, 4′, 5, 7-pentahydroxyflavone, C_15_H_10_O_7_) is an important flavonoid compound of natural origin, consisting of two aromatic A and B rings linked through the C ring with endocyclic oxygen atom and five hydroxyl groups attached to the 3, 3′, 4′, 5 and 7 positions. This molecule is found in many foods and plants, and is known to have a wide range of therapeutic properties, like an anti-oxidant, anti-toxic, anti-inflammatory etc. In this study for the first time we have revealed and investigated the pathways of the tautomeric transformations for the most stable conformers of the isolated quercetin molecule (Brovarets’ & Hovorun, 2019) *via* the intramolecular proton transfer. Energetic, structural, dynamical and polar characteristics of these transitions, in particular relative Gibbs free and electronic energies, characteristics of the intramolecular specific interactions–H-bonds and attractive van der Waals contacts, have been analysed in details. It was demonstrated that the most probable process among all investigated is the proton transfer from the O3H hydroxyl group of the C ring to the C2′ carbon atom of the C2′H group of the B ring along the intramolecular O3H…C2′ H-bond with the further formation of the C2′H_2_ group. It was established that the proton transfer from the hydroxyl groups to the carbon atoms of the neighboring CH groups is assisted at the transition states by the strong intramolecular HCH…O H-bond (~28.5 kcal∙mol^-1^). The least probable path of the proton transfer–from the C8H group to the endocyclic O1 oxygen atom–causes the decyclization of the C ring in some cases. It is shortly discussed the biological importance of the obtained results.

## Introduction

Quercetin molecule is a compound of natural origin, which is found in different foods and plants [1, 2]. This compound has attracted a lot of attention, since it is suggested to have a wide range of properties, such as an anti-oxidant, anti-toxic, anti-inflammatory etc. [3–20]. The structure of the quercetin contains two (A+C) and B rings and also has five hydroxyl groups at the 3, 3′, 4′, 5, 7 positions. So, due to these structural features it can acquire different conformations [21–26] and perform structural transitions between them due to the mutual rotations of the (A+C) and B rings around the C2-C1′ bond and also of the hydroxyl groups OH around the exocyclic C-O bonds [27]. Thus, investigations of the conformational transformations through the rotations around the C2-C1′ bond have been presented in literature [28–30]. In particular, in our recent works we have investigated in details conformational variety [26] and also conformational transitions of the quercetin molecule *via* the rotations of its rings [31] by using the quantum-mechanical (QM) calculations at the MP2/6-311++G(d,p)//B3LYP/6-311++G(d,p) level of theory and Bader’s quantum theory of “Atoms in Molecules” (QTAIM). Altogether, as a result of the study it was revealed 48 stable conformers (24 planar and 24 non-planar) with relative Gibbs free energies within the range of 0.0–25.3 kcal∙mol^-1^ under normal conditions, stabilized by the H-bonds (both classical OH…O and so-called unusual CH…O and OH…C) and attractive van der Waals contacts O…O, which have been divided into four different subfamilies by their structural properties [26]. Conformers of the quercetin molecule have been established to be polar structures with a dipole moment, which varies within the range from 0.35 to 9.87 Debay. We have also found out the interconversions of the 24 pairs of the conformers of the quercetin molecule *via* the rotation of its practicallay non-deformable (A+C) and B rings around the C2-C1' bond through the quasi-orthogonal transition states with Gibbs free energies of activation in the range of 2.17–5.68 kcal·mol^−1^ at normal conditions [31]. It was also provided comprehensive analysis of the 123 prototropic tautomers of the quercetin molecule [32, 33].

Also, quercetin can potentially acquire different prototropic tautomeric forms, but these data are weakly presented in the literature yet [34–38], despite the continuous comprehensive research of the quercetin molecule during the last decades [21–38].

It is widely known from the literature data that *proton transfer* is important biochemical phenomenon and plays important role in the biochemical reactions [39–45]. Thus, it was established that even movement of the single proton (SP) from the one site to another can cause significant changes of the energetic, structural and dynamical properties of the molecule, thus changing its functionality. In particular, tautomerization *via* the single (SPT) or double (DPT) proton transfer has been established for the canonical or non-canonical DNA base pairs [46–55], by the participation as of classical DNA bases [56], so by the participation of modified bases such as hypoxanthine [57–59], 5-bromouracil [60] and 2-aminopurine [61–64] molecules.

Currently, there are only some studies in the literature, devoted to the prototropic tautomerism of the quercetin molecule, in particular keto-enol tautomerism [34–38]. Their importance is caused by the relevance of the quercetin tautomers to the hydrogen↔deuterium (H↔D) exchange processes of its CH-groups [36], irreversible structural changes of the quercetin molecule at the increasing of the temperature [34–35] and tautomerization of the quercetin molecule at the transition to an excited electronic state [38]. However, possible ways of the formation of the rare prototropic tautomers of the quercetin molecule have not been carefully considered.

So, the aim of this study is to reveal and investigate the possible pathways of the prototropic transformations of the isolated quercetin molecule [65].

**Fig 1:**
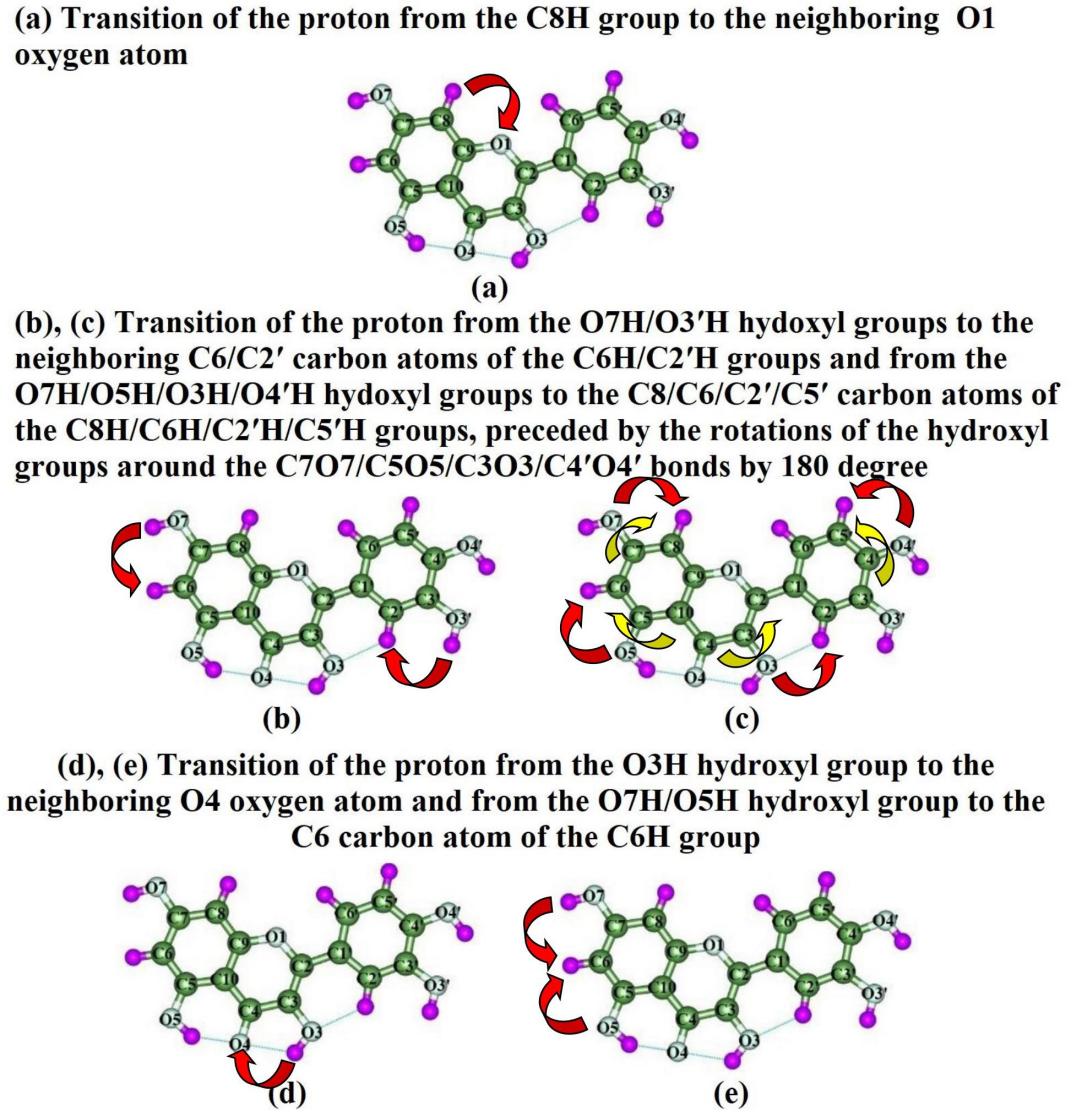
Schematic representations of the possible mechanisms of the intramolecular proton mobility in the quercetin molecule. Red arrows denote the directions of the proton transfer, yellow–rotations of the hydroxyl groups around the C-O bond by 180 degree.

As a result of this scrupulous investigation we have revealed possible ways of tautomerization of the quercetin molecule *via* the single proton transfer, which are entangled with the following phenomena (Fig 1):

- Proton transfer from the C8H group to the neighboring O1 oxygen atom.
- Transition of the proton from the O7H/O3′H hydroxyl groups to the carbon atoms of the neighboring C6H/C2′H groups.
- Migration of the proton from the O7H/O5H/O3H/O4′H hydroxyl groups to the carbon atoms of the neighboring C8H/C6H/C2′H/C5′H groups, preceded by the rotations of the hydroxyl groups around the C7O7/C5O5/C3O3/C4′O4′ bonds by 180 degree.
- Proton transfer from the O3H hydroxyl group to the neighboring O4 oxygen atom.
- Transition of the proton from the O7H/O5H hydroxyl groups to the C6 carbon atom of the neighboring C6H group.

Number of the important physico-chemical parameters of these transformations, in particular relative Gibbs free and electronic energies, characteristics of the intramolecular H-bonds and attractive van der Waals interactions have been analysed in details. Especial attention has been focused on the processes of the intramolecular tautomerization by proton transfer, which are more or less likely to occur. Possible chemical and biological roles of the obtained results have been shortly outlined.

## Computational methods

We have used the DFT B3LYP/6-311++G(d,p) level of theory [66–69], incorporated into Gaussian’09 program package [70], to provide the calculations of the geometrical structures and vibrational spectra of the prototropic tautomers of the quercetin molecule and transitions states (TSs) between them, which have been localized by Synchronous Transit-guided Quasi-Newton method [66]. This level of theory has been successfully approved for the calculations of the heterocyclic compounds [71–78]. Scaling factor of 0.9668 has been applied for the correction of the harmonic frequencies for the investigated structures [79, 80]. Electronic and Gibbs free energies under normal conditions have been calculated by single point calculations at the MP2/6-311++G(2df,pd) level of theory [81–83].

The Hessian-based predictor-corrector integration algorithm [84] has been applied for obtaining the IRC pathways in the forward and reverse directions from each TS.

The time *τ*_*99*.*9%*_, which is necessary to reach 99.9% of the equilibrium concentration of the reactant and product, the lifetime τ (1/*k*_*r*_) of the prototropic tautomers, the forward *k*_*f*_ and reverse *k*_*r*_ rate constants have been obtained by the well-known formulas of physico-chemical kinetics [85], respectively:

$$\tau_{99.9\%}=\frac{ln10^{3}}{k_{f}+k_{r}}$$ (1)

$$k_{f,r}=\Gamma \cdot \frac{k_{B}T}{h}e^{-\frac{\Delta \Delta G_{f,r}}{RT}}$$ (2)

where quantum tunneling effect has been accounted by Wigner’s tunneling correction Γ [86–88]:

$$\Gamma =1+\frac{1}{24}{\left(\frac{h\nu_{i}}{k_{B}T}\right)}^{2}$$ (3)

where *k*_*B*_−Boltzmann’s constant, *h*–Planck’s constant, *ΔΔG*_*f*,*r*_−Gibbs free energy of activation for the conformational transition in the forward (*f*) and reverse (*r*) directions, *ν*_*i*_−magnitude of the imaginary frequency associated with the vibrational mode at the TSs.

The distribution of the electron density has been analyzed by application of the program package AIM’2000 [89] with all default options and wave functions obtained at the B3LYP/6-311++G(d,p) level of theory for geometry optimisation. The presence of the (3,-1) bond critical point (BCP), bond path between hydrogen donor and acceptor and positive value of the Laplacian at this BCP (Δρ>0) have been considered as criteria for the formation of the H-bond and attractive van der Waals contact [62–63, 90–92].

Energies of the unusual intramolecular CH⋯O and OH⋯C H-bonds and attractive O⋯O and C⋯O van der Waals contacts have been obtained using Bader's quantum theory of Atoms in Molecules [93] by the empirical Espinosa-Molins-Lecomte (EML) formula [94, 95], based on the electron density distribution at the (3,-1) BCPs of the H-bonds:

$$E_{CH\cdots O/OH\cdots C/O\cdots O/C\cdots O}=0.5∙V(r)$$ (4)

where V(r)–value of a local potential energy at the (3,-1) BCP.

It should be noted, that for the CH…O H-bonds, which are strong with energy that exceeds 10 kcal∙mol^-1^, their energy have been estimated by the Brovarets’-Yurenko-Hovorun formula [96, 97], considered in the literature [98, 99]:

$$E_{CH\cdots O}=248.501∙\rho -0.367$$ (5)

The energies of the classical intramolecular OH⋯O H-bonds have been calculated by the Nikolaienko-Bulavin-Hovorun formula [100]:

$$E_{OH\cdots O}=-3.09+239∙\rho$$ (6)

where ρ–the electron density at the (3,-1) BCP of the H-bond.

All calculations have been performed for the tautomeric transitions of the quercetin molecule as their intrinsic property, that is adequate for modeling of the processes occurring in real systems [101–106].

In this work standard numeration of atoms has been used [2]. At this, prototropic tautomers of the quercetin molecule have been designated by the asterisk; subscript corresponds to the localization of the mobile protons. Numeration of the conformers (highlighted in bold) is the same, as in the previous work [26].

## Results and discussion

In the process of this study we have suggested different ways of the formation of the prototropic tautomers of the most stable conformer **1** [26] of the quercetin molecule. Then, by using the method of “trials and errors” we have localized TSs for these tautomeric transformations, occurring *via* the intramolecular proton transfer. However, only some of the suggested tautomeric transformations have been confirmed, while others of them have been modified in the course of the investigation.

**Fig 2:**
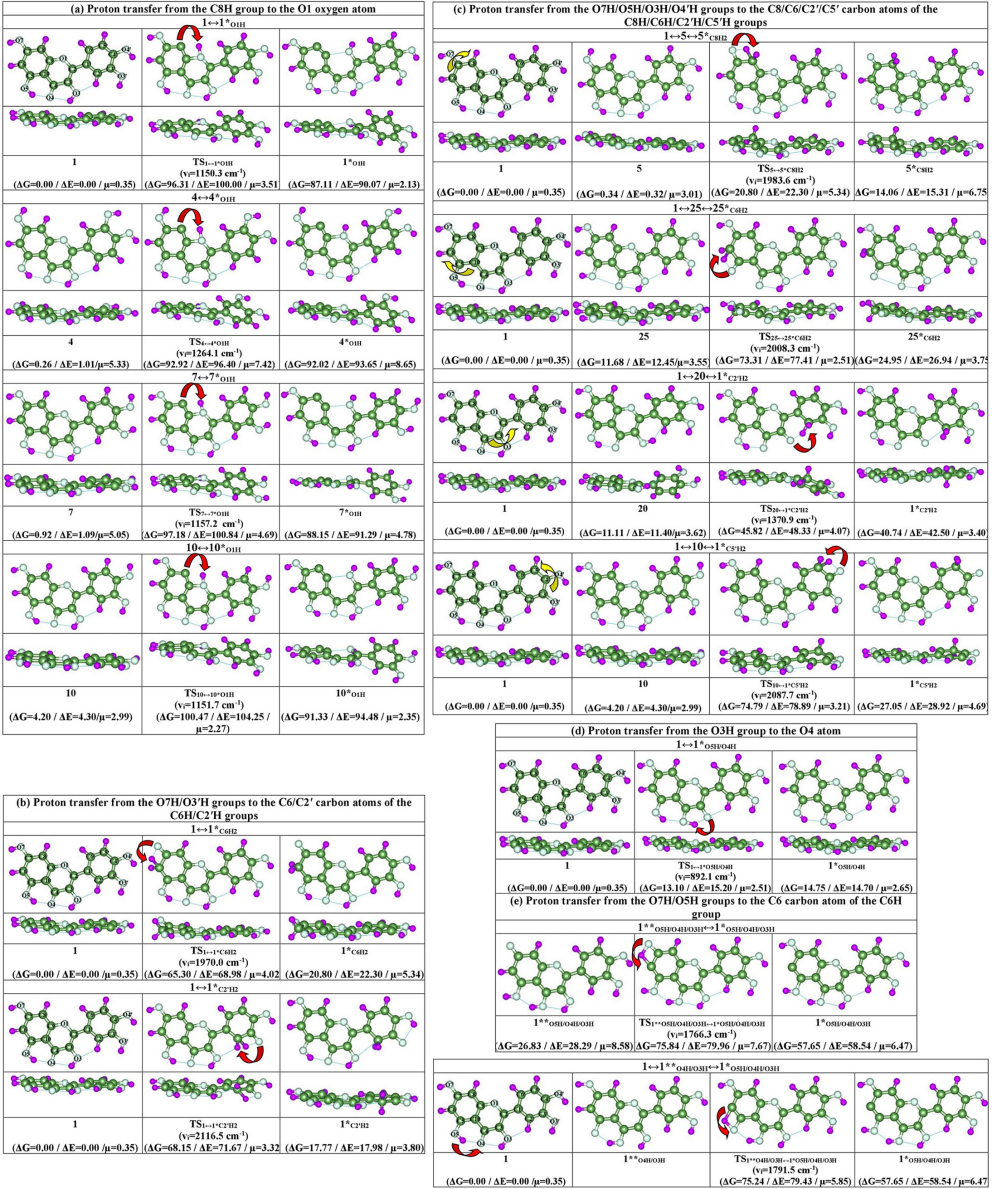
Reaction pathways for the intramolecular proton transfer in the isolated quercetin molecule; initial and terminal states with TSs between them have been obtained at the MP2/6-311++G(2df,pd) // B3LYP/6-311++G(d,p) level of QM theory (low index near formed tautomers denotes the site of the localization of the transferred proton). Gibbs free ΔG and electronic ΔE energies (kcal∙mol^-1^), imaginary frequencies v_i_ at the TS and dipole moments μ (Debay) are provided below reaction paths. Dotted lines indicate intramolecular specific interactions. Red arrows denote the intramolecular transition of the proton, while yellow arrows–rotations of the hydroxyl groups. See also Tables 1 and 2.

So, in this study we have considered the following mechanisms of the tautomerization of the quercetin molecule, in particular of the most stable conformer **1**, that can proceed in the different ways through the intramolecular proton transfer (see Figs 1 and 2, Tables 1 and 2).

**Table 1:** Energetic and kinetic characteristics of the tautomeric transformations by the intramolecular proton transfer in the isolated quercetin molecule obtained at the MP2/6-311++G(2df,pd)//B3LYP/6-311++G(d,p) level of QM theory under normal conditions (see also Fig 1).

Tautomeric transition	ν_i_^a^	ΔG^b^	ΔE^c^	ΔΔG_TS_^d^	ΔΔE_TS_^e^	ΔΔG^f^	ΔΔE^g^	k_f_^h^	k_r_^i^	τ_99.9%_^j^	τ^k^
**(a) Proton transfer from the C8H group to the O1 oxygen atom**											
1↔1*_O1H_	1150.3	87.11	90.07	96.31	100.00	9.20	9.93	3.09∙10^−58^	2.43∙10^6^	2.84∙10^−6^	4.12∙10^−7^
4↔4*_O1H_	1264.1	91.76	92.64	92.66	95.39	0.90	2.75	1.64∙10^−55^	3.31∙10^12^	2.09∙10^−12^	3.02∙10^−13^
7↔7*_O1H_	1157.2	87.23	90.20	96.26	99.75	9.03	9.55	3.58∙10^−58^	3.26∙10^6^	2.12∙10^−6^	3.07∙10^−7^
10↔10*_O1H_	1151.7	87.13	90.18	96.27	99.95	9.14	9.77	3.31∙10^−58^	2.69∙10^6^	2.57∙10^−6^	3.72∙10^−7^
**(b) Proton transfer from the O7H/O3′H groups to the C6/C2′ carbon atoms of the C6H/C2′H groups**											
1↔1*_C6H2_	1970.0	20.80	22.30	65.30	68.98	44.50	46.68	3.53∙10^−35^	6.37∙10^−20^	1.08∙10^20^	1.57∙10^19^
1↔1*_C2'H2_	2116.5	17.77	17.98	68.15	71.67	50.38	53.69	3.21∙10^−37^	3.47∙10^−24^	1.99∙10^24^	2.88∙10^23^
**(c) Proton transfer from the O7H/O5H/O3H/O4′H groups to the C8/C6/C2′/C5′ carbon atoms of the C8H/C6H/C2′H/C5′H groups**											
5↔5*_C8H2_	1983.6	13.72	14.99	20.46	21.98	6.74	6.99	2.77∙10^−2^	3.21∙10^8^	2.15∙10^−8^	3.12∙10^−9^
25↔25*_C6H2_	2008.3	13.27	14.49	61.63	64.96	48.36	50.47	1.79∙10^−32^	9.69∙10^−23^	7.13∙10^22^	1.03∙10^22^
20↔1**_C2'H2_	1370.9	29.63	31.10	34.71	36.93	5.08	5.83	5.79∙10^−13^	3.14∙10^9^	2.20∙10^−9^	3.19∙10^−10^
10↔1*_C5'H2_	2087.7	22.85	24.62	70.59	74.59	47.74	49.97	5.09∙10^−39^	2.93∙10^−22^	2.35∙10^22^	3.41∙10^21^
**(d) Proton transfer from the O3H group to the O4 atom**											
1↔1*_O5H/O4H_	892.1	14.30	14.70	13.10	15.20	-1.20	0.50	2.62∙10^3^	8.09∙10^13^	8.54∙10^−14^	1.24∙10^−14^
**(e) Proton transfer from the O7H/O5H groups to the C6 carbon atom of the C6H group**											
1**_O5H/O4H/O3H_↔1*_O5H/O4H/O3H_	1766.3	30.82	30.25	49.01	51.67	18.19	21.42	2.66∙10^−23^	1.07	6.43	0.93
1**_O4H/O3H_**↔** 1*_O5H/O4H/O3H_	1791.5	57.65	58.54	75.24	79.43	17.59	20.89	1.56∙10^−42^	3.02	2.29	0.33

^a^The imaginary frequency at the TS of the tautomeric transition, cm^-1^.

^b^The Gibbs free energy of the initial relatively the terminal structure of the tautomerisation reaction (T = 298.15 K), kcal∙mol^-1^.

^c^The electronic energy of the initial relatively the terminal structure of the tautomerisation reaction, kcal∙mol^-1^.

^d^The Gibbs free energy barrier for the forward tautomerisation reaction, kcal∙mol^-1^.

^e^The electronic energy barrier for the forward tautomerisation reaction, kcal∙mol^-1^.

^f^The Gibbs free energy barrier for the reverse tautomerisation reaction, kcal∙mol^-1^.

^g^The electronic energy barrier for the reverse tautomerisation reaction, kcal∙mol^-1^.

^h^The rate constant for the forward tautomerisation reaction, s^-1^.

^i^The rate constant for the reverse tautomerisation reaction, s^-1^.

^j^The time necessary to reach 99.9% of the equilibrium concentration between the reactant and the product of the tautomerisation reaction, s.

^k^The lifetime of the product of the tautomerisation reaction, s.

**Table 2:** Energetical, electron-topological and geometrical characteristics of the intramolecular specific contacts–H-bonds and attractive van der Waals (vdW) contacts, polar and geometrical parameters of the investigated structures of the isolated quercetin molecule obtained at the B3LYP/6-311++G(d,p) level of QM theory (see also Fig 1).

Conformer, TS and tautomer	AH⋯B H-bond / A⋯B vdW contact	*E*_*AH⋯B *_*/ E*_*A··B *_^a^	*ρ*^b^	*Δρ*^c^	*100∙ε*^d^	*d*_*A⋯B*_^e^	*d*_*H⋯B*_^f^	∠AH⋯B^g^	ΔC3-C2-C1′-C6′^h^	μ^i^
**(a) Transition of the proton from the C8H group to the O1 oxygen atom**										
1↔1*_O1H_										
**1**	O5H . . .O4	6.71	0.041	0.124	1.52	2.655	1.770	147.3	180.0	0.35
	O3H . . .O4	3.36	0.027	0.103	60.55	2.625	2.009	119.0		
	C2'H . . .O3	4.01*	0.018	0.076	0.92	2.883	2.137	123.8		
**TS**_1↔1*O1H_	O5H . . .O4	2.89	0.025	0.082	1.10	2.830	1.990	142.9	-164.8	3.38
	O3H . . .O4	3.84	0.029	0.109	38.00	2.600	1.963	120.4		
	C2'H . . .O3	3.32*	0.016	0.063	10.19	2.924	2.221	120.6		
1*_O1H_ **[32]**	O5H . . .O4	9.10	0.051	0.148	2.33	2.565	1.672	147.8	-155.3	1.83
	O3H . . .O4	5.75	0.037	0.134	17.66	2.509	1.853	121.6		
	C9 . . .O1	6.47*	0.029	0.090	2.46	2.895	-	-		
	C2'H . . .O3	3.38*	0.016	0.064	26.96	2.856	2.253	113.1		
4↔4*_O1H_										
**4**	O5H . . .O4	6.47	0.040	0.124	1.46	2.659	1.776	147.3	180.0	5.33
	O3H . . .O4	3.36	0.027	0.103	60.63	2.624	2.009	118.9		
	C6'H . . .O3	3.83*	0.018	0.073	0.80	2.895	2.159	123.3		
**TS**_4↔4*O1H_	O5H . . .O4	2.65	0.024	0.081	1.20	2.835	1.995	143.0	162.2	7.77
	O3H . . .O4	3.84	0.029	0.109	38.52	2.601	1.965	120.2		
	C6'H . . .O3	3.07*	0.015	0.058	12.45	2.942	2.261	119.2		
4*_O1H_ **[32]**	O5H . . .O4	5.04	0.034	0.112	1.32	2.705	1.842	145.2	154.4	8.88
	O3H . . .O4	4.32	0.031	0.113	30.34	2.579	1.939	120.5		
	C6'H . . .O3	2.57*	0.012	0.048	22.18	2.975	2.358	114.7		
7↔7*_O1H_										
**7**	O5H . . .O4	6.71	0.041	0.125	1.50	2.652	1.767	147.3	180.0	5.05
	O3H . . .O4	3.12	0.026	0.102	68.01	2.630	2.020	118.5		
	C2'H . . .O3	3.83*	0.018	0.073	0.02	2.886	2.160	122.4		
**TS**_7↔7*O1H_	O5H . . .O4	2.89	0.025	0.082	1.09	2.828	1.988	142.9	-162.0	4.84
	O3H . . .O4	3.60	0.028	0.107	42.14	2.606	1.977	119.8		
	C2'H . . .O3	3.03*	0.014	0.058	13.43	2.936	2.267	118.2		
7*_O1H_ **[32]**	O5H . . .O4	9.10	0.051	0.147	2.27	2.565	1.673	147.8	-151.5	4.65
	O3H . . .O4	5.28	0.035	0.131	19.50	2.516	1.869	121.0		
	C9 . . .O1	6.54*	0.029	0.091	2.45	2.374	-	-		
	C2'H . . .O3	2.98*	0.014	0.056	36.27	2.876	2.327	109.7		
10↔10*_O1H_										
**10**	O5H . . .O4	6.71	0.041	0.124	1.51	2.654	1.770	147.3	180.0	2.99
	O3H . . .O4	3.12	0.026	0.103	61.90	2.626	2.011	118.9		
	C2'H . . .O3	3.98*	0.018	0.075	1.02	2.889	2.141	124.1		
**TS**_10↔10*O1H_	O5H . . .O4	2.89	0.025	0.082	1.09	2.830	1.990	142.9	-164.7	2.34
	O3H . . .O4	3.84	0.029	0.109	38.76	2.601	1.966	120.3		
	C2'H . . .O3	3.27*	0.015	0.062	10.11	2.932	2.227	120.8		
10*_O1H_ **[32]**	O5H . . .O4	9.10	0.051	0.148	2.31	2.565	1.673	147.8	-154.8	2.24
	O3H . . .O4	5.51	0.036	0.133	18.06	2.510	1.857	121.5		
	C9 . . .O1	6.52*	0.029	0.091	2.51	2.375	-	-		
	C2'H . . .O3	3.30*	0.015	0.062	27.26	2.865	2.263	113.1		
**(b) Transition of the proton from the O7H/O3′H groups to the neighboring C6/C2′ carbon atoms of the C6H/C2′H groups**										
1↔1*_C6H2_										
**TS**_1↔1*C6H2_	HC6H . . .O7	28.46**	0.116	0.059	20.81	2.211	1.388	105.3	179.3	3.96
	O5H . . .O4	8.62	0.049	0.136	1.51	2.595	1.692	148.4		
	O3H . . .O4	3.12	0.026	0.101	69.93	2.633	2.025	118.4		
	C2'H . . .O3	3.97*	0.018	0.075	1.12	2.886	2.141	123.8		
1*_C6H2_ **[32]**	O5H . . .O4	8.86	0.050	0.137	1.47	2.587	1.687	147.5	180.0	5.34
	O3H . . .O4	2.65	0.024	0.099	96.94	2.646	2.048	117.7		
	C2'H . . .O3	4.14*	0.019	0.078	1.09	2.872	2.126	123.9		
1↔1*_C2'H2_										
**TS**_1↔1*C2'H2_	O5H . . .O4	6.47	0.040	0.124	1.37	2.657	1.775	147.0	-176.8	3.94
	O3H . . .O4	2.89	0.025	0.100	76.39	2.637	2.032	118.1		
	C2'H . . .O3	3.05*	0.014	0.058	39.78	2.833	2.322	106.5		
	HC2'H . . .O3'	26.97**	0.110	0.072	28.82	2.244	1.409	105.0		
1*_C2'H2_ **[32]**	O5H . . .O4	6.47	0.040	0.124	1.43	2.657	1.775	147.1	180.0	3.80
	O3H . . .O4	3.12	0.026	0.102	68.85	2.631	2.022	118.4		
	O3 . . .C2'	2.90*	0.012	0.056	374.04	2.807	-	-		
	O4'H . . .O3'	2.65	0.024	0.098	139.26	2.650	2.071	116.2		
**(c) Transition of the proton from the O7H/O5H/O3H/O4′H groups to the carbon atoms of the C8H/C6H/C2′H/C5′H groups, preceded by the rotations of the hydroxyl groups around the C7O7/C5O5/C3O3/C4′O4′ axes by 180 degree**										
5↔5*_C8H2_										
**5**	O5H . . .O4	6.47	0.040	0.123	1.47	2.660	1.777	147.2	180.0	3.01
	O3H . . .O4	3.36	0.027	0.104	58.22	2.623	2.004	119.1		
	C2'H . . .O3	4.01*	0.018	0.076	0.91	2.883	2.138	123.8		
**TS**_5↔5*C8H2_	O5H . . .O4	8.35	0.048	0.136	1.28	2.607	1.700	149.6	177.3	4.60
	O3H . . .O4	6.78	0.026	0.103	61.23	2.623	2.010	118.6		
	C2'H . . .O3	3.87*	0.018	0.073	1.71	2.894	2.151	123.6		
	HC8H . . .O7	30.20**	0.123	0.033	17.10	2.209	1.363	105.4		
5*_C8H2_ **[32]**	O5H . . .O4	7.19	0.043	0.130	1.07	2.642	1.746	149.1	180.0	6.69
	O3H . . .O4	3.36	0.027	0.104	62.47	2.620	2.008	118.5		
	C2'H . . .O3	3.84*	0.018	0.073	0.71	2.897	2.154	123.7		
25↔25*_C6H2_										
**25**	O3H . . .O4	4.56	0.032	0.117	31.44	2.571	1.921	121.2	180.0	3.55
	O5 . . .O4	2.91*	0.012	0.049	13.37	2.765	-	-		
	C2'H . . .O3	3.93*	0.018	0.074	0.62	2.890	2.145	123.8		
**TS**_25↔25*C6H2_	HC6H . . .O5	31.44**	0.128	0.012	15.09	2.208	1.347	105.3	179.7	2.51
	O3H . . .O4	4.16	0.030	0.112	2.00	2.590	1.950	120.5		
	C2'H . . .O3	3.87*	0.018	0.073	0.56	2.895	2.151	123.8		
25*_C6H2_ **[32]**	O5 . . .O4	2.55*	0.010	0.041	188.30	2.903	-	-	179.6	4.46
	O3H . . .O4	4.80	0.033	0.118	28.83	2.564	1.909	121.5		
	C2'H . . .O3	3.75*	0.017	0.071	0.38	2.906	2.162	123.8		
20↔1**_C2'H2_										
**20**	O5H . . .O4	8.38	0.048	0.136	1.36	2.600	1.700	148.6	135.5	3.62
	O3H . . .C2'	2.36*	0.012	0.045	251.5	3.033	2.242	138.5		
**TS**_20↔1**C2'H2_	O5H . . .O4	8.71	0.049	0.138	1.11	2.591	1.692	148.5	148.4	4.07
	HC2'H . . .O3	27.22**	0.111	0.085	3.56	2.509	1.398	141.0		
1**_C2'H2_ **[32]**	O5H . . .O4	8.62	0.049	0.138	0.89	2.594	1.697	148.2	180.0	3.40
	C2' . . .O3	4.47*	0.018	0.075	458.16	2.695	-	-		
10↔1*_C5'H2_										
**TS**_10↔1*C5'H2_	O5H . . .O4	6.47	0.040	0.124	1.48	2.657	1.774	147.1	-175.7	3.21
	O3H . . .O4	3.36	0.027	0.104	57.17	2.621	2.003	119.1		
	C2'H . . .O3	4.28*	0.019	0.080	2.57	2.885	2.107	126.5		
	HC5'H . . .O4'	27.47**	0.112	0.070	25.78	2.232	1.407	104.6		
1*_C5'H2_ **[32]**	O5H . . .O4	6.47	0.040	0.123	1.37	2.661	1.780	146.8	-173.4	4.69
	O3H . . .O4	3.36	0.027	0.106	52.39	2.614	1.994	119.2		
	C5'H . . .O4'	4.11*	0.019	0.077	3.93	2.894	2.125	125.8		
**(d) Transition of the proton from the O3H group to the O4 oxygen atom**										
1↔1*_O5H/O4H_										
**TS**_1↔1*O5H/O4H_	O5H . . .O4	2.65	0.024	0.090	0.90	2.802	1.964	143.0	180.0	3.79
	O4H . . .O3	22.48	0.107	0.082	1.04	2.354	1.408	136.1		
	C2'H . . .O3	3.75*	0.018	0.066	3.88	2.958	2.175	127.2		
1*_O5H/O4H_ **[32]**	O5H . . .O4	2.89	0.025	0.100	4.07	2.754	1.923	142.1	180.0	4.06
	O4H . . .O3	7.43	0.044	0.128	12.54	2.503	1.783	125.2		
	C2'H . . .O3	4.53*	0.021	0.077	3.16	2.903	2.114	127.3		
**(e) Transition of the proton from the O7H group to the C6 carbon atom of the C6H group**										
1**_O5H/O4H/O3H_↔1*_O5H/O4H/O3H_										
1**_O5H/O4H/O3H_ **[32]**	O4H . . .O5	5.04	0.034	0.128	6.17	2.634	1.806	140.7	180.0	8.58
	O3H . . .O4	2.17	0.022	0.100	66.71	2.613	2.047	115.4		
	C2'H . . .O3	4.15*	0.019	0.078	1.36	2.868	2.130	123.2		
**TS**_1**O5H/O4H/O3H↔_ _1*O5H/O4H/O3H_	O4H . . .O5	5.75	0.037	0.135	6.00	2.613	1.765	142.5	180.0	7.99
	O3H . . .O4	2.17	0.022	0.099	79.70	2.617	2.055	115.1		
	C2'H . . .O3	4.11*	0.019	0.077	1.28	2.871	2.133	123.2		
1*_O5H/O4H/O3H_ **[32]**	O4H . . .O5	6.47	0.040	0.138	5.72	2.599	1.740	143.4	180.0	6.68
	O3H . . .O4	1.93	0.021	0.097	126.99	2.633	2.076	114.7		
	C2'H . . .O3	4.21*	0.019	0.079	1.29	2.863	2.124	123.3		
1↔1*_O5H/O4H/O3H_										
**TS**_1**O4H/O3H↔_ _1*O5H/O4H/O3H_	O4H . . .O5	4.47	0.032	0.100	1.67	2.735	1.856	145.7	180.0	6.05
	C2'H . . .O3	4.32*	0.019	0.081	1.39	2.686	2.143	113.9		
1*_O5H/O4H/O3H_ **[32]**	O4H . . .O5	6.47	0.040	0.138	5.72	2.599	1.740	143.4	180.0	6.68
	O3H . . .O4	1.93	0.021	0.097	126.99	2.633	2.076	114.7		
	C2'H . . .O3	4.21*	0.019	0.079	1.29	2.863	2.124	123.3		

^a^The energy of the AH⋯B / A⋯B specific contact, calculated by Espinose-Molins-Lecomte [94, 95] (marked with an asterisk), Brovarets-Yurenko-Hovorun [96] (marked with a double asterisk) or Nikolaienko-Bulavin-Hovorun [100] formulas, kcal∙mol^-1^

^b^The electron density at the (3,-1) BCP of the specific contact, a.u.

^c^The Laplacian of the electron density at the (3,-1) BCP of the specific contact, a.u.

^d^The ellipticity at the (3,-1) BCP of the specific contact

^e^The distance between the A and B atoms of the AH⋯B / A⋯B specific contact, Å

^f^The distance between the H and B atoms of the AH⋯B H-bond, Å

^g^The H-bond angle, degree

^h^The dihedral angle **∠**C3-C2-C1′-C6′, degree

^i^The dipole moment of the molecule, Debay. See also Fig 1 and Table 1.

It was established that these transformations of the quercetin molecule are accompanied by the changes of their geometry, dipole moment rearrangement and breakage and formation of the intramolecular specific contacts (H-bonds and attractive van der Waals contacts).

Analysis of the investigated mechanisms and their discussion are provided further one-by-one.

**a) Proton transfer from the C8H group to the O1 atom.** First of the considered mechanisms consists in the intramolecular transition of the proton, localized at the C8 carbon atom, to the neighboring endocyclic oxygen atom O1, leading to the formation of the new tautomer with formed O1H hydroxyl group (Figs 1 and 2). We have analysed this tranformation for the case of the main stable conformer **1** of the quercetin molecule and also checked it for the others–conformers **4**, **7** and 10: 1↔1*_O1H_ (ΔΔG_TS_ = 96.31); **4**↔4*_O1H_ (ΔΔG_TS_ = 92.66); **7**↔7*_O1H_ (ΔΔG_TS_ = 96.26) and **10**↔10*_O1H_ (ΔΔG_TS_ = 96.27 kcal∙mol^-1^) (Table 1).

Finally, four new prototropic tautomers have been formed– 1*_O1H_ (ΔG = 9.20), 4*_O1H_ (ΔG = 0.90), 7*_O1H_ (ΔG = 9.03) and 10*_O1H_ (ΔG = 9.14 kcal∙mol^-1^) (Table 1). Notably, all of them, except the case of the conformer **4**, which contains opened C-ring and new exotic strong attractive van der Waals contact C9 o…O1 (~6.5 kcal∙mol^-1^ (Table 2)) instead of the C9-O1 covalent bond in the C ring. In the case of the **4**↔4*_O1H_ tautomeric transition, the covalent bond C9-O1 survives during this transformation. Notably, three lower H-bonds, stabilizing conformers–O5H …O4, O3H …O4 and C2'H …O3,–remain the same, changing only their energies during tautomerisation (Table 2).

The **1**↔1*_O1H_ tautomerisation reaction occurs *via* quite high activation barrier and TS_1↔1*O1H_ with high imaginary frequency (v_i_ = 1150.3 cm^-1^). Notably, that we have checked and revealed that this transition is typical for all investigated conformers. Thus, the Gibbs free energies of activation consist ~93–96 kcal∙mol^-1^ for the **4**↔4*_O1H_, **7**↔7*_O1H_ and **10**↔10*_O1H_ tautomeric transformations of the non-planar conformers **4**, **7** and **10** (see Fig 1 and Table 1).

At this, the tautomer 4*_O1H_ has been established to be dynamically-unstable (ΔΔG = 0.9 kcal∙mol^-1^)–its lifetime τ = 3∙10^−13^ s (Table 1) is less than the period of the most low-frequency torsional vibration of the rings around the C2-C1′ bond, which could not develop during this lifetime.

We have also tried to localize the tautomer with the proton, transferred to the O1 oxygen atom from the other neighboring C6H group for others conformers of the quercetin molecule [26] in the case, when these groups are closely located. However, since the stable structure could not be localized, that means that in fact this reaction would not occur.

So, intramolecular proton transfer from the C8H group to O1 oxygen atom causes decyclization (opening) of the C ring of the quercetin molecule. We consider this result quite important, taking into account how much attention attracts prototropic, in particular ring-chain tautomerism [107, 108], in the modern computer-aided drug design [42, 43].

**b) Transition of the proton from the O7H/O3′H hydroxyl groups to the carbon atoms of the neighboring C6H/C2′H groups.** Firstly, we have considered all possible sites for the proton transfer from the hydroxyl groups to the carbon atoms of the neighboring CH groups with the formation of the CH_2_ group. It was revealed only two tautomerization reactions, which occur in this case–O7H→C6H and O3′H→C2′H. Investigated tautomeric transformations– **1**↔1*_C6H2_ (ΔΔG_TS_ = 65.30) and **1**↔1*_C2'H2_ (ΔΔG_TS_ = 68.15 kcal∙mol^-1^)–occur *via* the intramolecular proton transfer, which are preceded by the rotations of the hydroxyl groups to the CH groups, with Gibbs free energy barriers of activation– 65.30 and 68.15 kcal∙mol^-1^, respectively. As a result of these tautomerisations, the planar tautomers 1*_C6H2_ and 1*_C2'H2'_ with relative Gibbs free energies 44.50 and 50.38 kcal∙mol^-1^, containing the C6H_2_ and C2′H_2_ groups have been formed, respectively (Fig 1, Tables 1 and 2).

These processes of tautomerisation are assisted by the strong intramolecular HC6H …O7 (28.46) and HC2'H …O3' (26.97 kcal∙mol^-1^) H-bonds at the TS_1↔1*C6H2_ and TS_1↔1*C2'H2_ transition states. All others H-bonds (O5H …O4, O3H …O4 and C2'H …O3) remain the same at the starting **1** and terminal 1*_C6H2_ structures for the transformation **1**↔1*_C6H2_, while the initial set of the H-bonds (O5H …O4, O3H …O4, C2'H …O3) rearranges into the terminal network of the H-bonds (O5H …O4, O3H …O4, O3 …C2', O4'H …O3') for the transformation **1**↔1*_C2'H2_ (see Fig 1 and Table 2).

**c) Transitions of the proton from the O7H/O5H/O3H/O4′H hydroxyl groups to the C8/C6/C2′/C5′ carbon atoms of the C8H/C6H/C2′H/C5′H groups, which are preceded by the rotations of the hydroxyl groups around the C7O7/C5O5/C3O3/C4′O4′ bonds by 180 degree.** We have also surveyed other sites of the proton attachment for the possibility of the proton transfer to them. However, analysed sites require rotation of the OH hydroxyl groups around the C-O bond by 180 degree, leading to the prototropic transformations–O7H→C8H, O5H→C6H, O3H→C2′H and O4′H→C5′H. Only in this way of the initial rotation of the OH hydroxyl group of the basic tautomer **1** of the quercetin molecule [26], it is possible to form new prototropic tautomers through the intramolecular transfer of single proton. However, precise investigation of the transformations *via* the rotations of the OH hydroxyl groups would be the subject of the next study [27], since in this paper we are focusing exactly on the mechanisms of the intramolecular proton transfer.

Thus, it was revealed the following chains of the SPT reactions (Fig 1, Table 1): 5↔5*_C8H2_ (ΔΔG_TS_ = 20.46); 25↔25*_C6H2_ (ΔΔG_TS_ = 61.63); 20↔1**_C2'H2_ (ΔΔG_TS_ = 34.71) and 10↔1*_C5'H2_ (ΔΔG_TS_ = 70.59 kcal∙mol^-1^).

Notably, all of these reactions are assisted by the formation at the TSs of the extremely strong intramolecular HCH…O H-bond (26.97–31.44 kcal∙mol^-1^ (Table 2)) between the CH_2_ group and neighboring oxygen atom. At this, all other H-bonds remain practically unchanged at the initial and terminal states (Fig 1, Table 2). Prototropic tautomers, which are formed in this case, are planar structures (Table 2).

Notably, activation barriers for the considered **5**↔5*_C8H2_, **25**↔25*_C6H2_ and **10**↔1*_C5'H2_ tautomerisations are quite high (~62–71 kcal∙mol^-1^), except the cases **5**↔5*_C8H2_ (ΔG = 20.46) and **20**↔1**_C2'H2_ (ΔG = 34.71 kcal∙mol^-1^). This relatively small value of the barrier can be explained by the formation of the six-membered ring at the TS_20↔1**C2'H2_ and by moving of the proton along the O3H…C2′ H-bond [26]. In those cases, when TS_25↔25*C6H2_, TS_5↔5*C8H2_ and TS_10↔1*C5'H2_ contains four-membered rings and proton does not move along the intramolecular H-bond–the values of the activation barriers are much higher. At this, 1**_C2'H2_ tautomer is the only one structure, which has the C2 = C1′ double bond.

d) Transition of the proton from the O3H hydroxyl group to the O4 atom.

Further we investigated structural mechanisms of the single proton transfer, occurring between the O5H and O3H hydroxyl groups. Thus, it was found that proton can transfer from the O3H hydroxyl group to the O4 oxygen atom through the 1↔1*_O5H/O4H_ tautomerization reaction with the barrier ΔΔG_TS_ = 13.10 kcal∙mol^-1^. However, terminal localized complex is dynamically unstable–reverse Gibbs free energy barrier has negative value (ΔΔG = -1.20 kcal∙mol^-1^) (exactly in this case it is observed at TSs the lowest value of the imaginary frequency ν_i_ = 892.1 cm^-1^) (Table 1).

It is logically to think by analogy that the same intramolecular proton transfer should occur from the O5H hydroxyl group to the O4 oxygen atom. But in this case the TSs and tautomers could not be localized at all.

e) Proton migration from the O7H/O5H hydroxyl groups to the C6 atom of the C6H group.

We also considered tautomeric transformation of the 1*_O5H/O4H/O3H_ tautomer by the transition of the protons from the O7H/O5H hydroxyl groups to the neighboring C6 atom of the C6H group.

Thus, in the first case the 1**_O5H/O4H/O3H_↔1*_O5H/O4H/O3H_ tautomerization reaction proceeds *via* the transfer of proton from the O7H hydroxyl group to the neighboring C6 atom and occurs *via* the quite high barrier (ΔΔG_TS_ = 49.01 kcal∙mol^-1^) and leads to the dynamically stable tautomer 1*_O5H/O4H/O3H_ (Fig 1, Table 1).

In the second case, the intramolecular proton transfer in the 1**_O4H/O3H_ tautomer from the O5H hydroxyl group to the neighboring C6 carbon atom of the C6H group– 1**_O4H/O3H_↔1*_O5H/O4H/O3H_ –occurs through the TS_1**O4H/O3H↔1*O5H/O4H/O3H_ (ΔΔG_TS_ = 75.24 kcal∙mol^-1^) and leads to the formation of the dynamically unstable 1**_O4H/O3H_ tautomer with relative electronic energy 14.87 kcal∙mol^-1^, which further causes chain transfer of the proton from the O4H hydroxyl group to the O5 oxygen atom, leading to the stable conformer **1** (Fig 1, Table 1).

It can be expected the reduction of the values of the activation barriers at the consideration of these transitions in the polar solutions or assisted by various ligands.

## Conclusions and perspectives

Presented QM/QTAIM computational modeling of the tautomers formation through the intramolecular proton transfer shows that the quercetin molecule is able to tautomerise *via* the different routes within the framework of the classical valency rules:

- Proton transfer from the C8H group to the O1 atom, leading in three cases to the breakage of the C ring: **1**↔1*_O1H_, **7**↔7*_O1H_ and **10**↔10*_O1H_, except the case of **4**↔4*_O1H_ reaction (ΔΔG_TS_ ~ 93–96 kcal∙mol^-1^).
- Transition of the proton from the O7H/O3′H hydroxyl groups to the carbon atoms of the neighboring C6H/C2′H groups: **1**↔1*_C6H2_ and **1**↔1*_C2'H2_ (ΔΔG_TS_ ~ 65–68 kcal∙mol^-1^).
- Migration of the proton from the O7H/O5H/O3H/O4′H hydroxyl groups to the carbon atoms of the C8H/C6H/C2′H/C5′H groups, preceded by the rotations of the hydroxyl groups around the C7O7/C5O5/C3O3/C4′O4′ bond by 180 degree: 5↔5*_C8H2_ (ΔΔG_TS_ = 20.46); 25↔25*_C6H2_ (ΔΔG_TS_ = 61.63); 20↔1**_C2'H2_ (ΔΔG_TS_ = 34.71) and 10↔1*_C5'H2_ (ΔΔG_TS_ = 70.59 kcal∙mol^-1^).
- Proton transfer from the O3H hydroxyl group to the O4 oxygen atom with the formation of the dynamically-unstable tautomer: **1**↔1*_O5H/O4H_ (ΔΔG_TS_ ~ 13 kcal∙mol^-1^).
- Transition of the proton from the O7H/O5H hydroxyl group to the C6 carbon atom of the C6H group: 1**_O5H/O4H/O3H_↔1*_O5H/O4H/O3H_ and 1**_O4H/O3H_↔1*_O5H/O4H/O3H_ (ΔΔG_TS_ ~ 49–75 kcal∙mol^-1^).

These prototropic transformations of the quercetin molecule are accompanied by the geometrical changes, dipole moment rearrangement and breakage or formation of the intramolecular specific contacts (H-bonds and attractive van der Waals contacts).

It was demonstrated that the most probable process among all investigated is the proton transfer from the O3H hydroxyl group to the C2′ carbon atom of the C2′H of the B ring along the intramolecular O3H…C2′ H-bond with the further formation of the C2′H_2_ group, while the least probable proton transfer occurs from the C8H group to the O1 oxygen atom–causes the decyclization of the C ring.

Obtained results can be useful for the planning of targeted chemical experiments, aimed at the acceleration of the reaction of intramolecular tautomerization of a quercetin molecule by the ligands of different structure and origin, as well as for the better understanding of the mechanisms of the course of reactions, related to the metabolism of the quercetin molecule.

## References

[1] Vermerris W., Nicholson R. Phenolic compound biochemistry Dordrecht: Springer, 2006, 275 p.

[2] Andersen M., Markham K.R. Flavonoids: chemistry, biochemistry, and applications. New York: CRC Press, 2006, 1197 p.

[3] Sharma A., Kashyap D., Sak K., Tuli H.S., Sharma A.K. Therapeutic charm of quercetin and its derivatives: a review of research and patents. Pharm. Pat. Anal., 2018, 7, 15–32. 10.4155/ppa-2017-0030 29227203

[4] Li Y., Yao J., Han Ch, Yang J., Chaudhry M.T., Wang Sh., Liu H., Yin Y. Quercetin, inflammation and immunity. Nutrients, 2016, 8, 167 10.3390/nu8030167 26999194 PMC4808895

[5] Amić D., Stepanić V., Lučić B., Marković Z., Dimitrić Marković J.M. PM6 study of free radical scavenging mechanisms of flavonoids: why does O-H bond dissociation enthalpy effectively represent free radical scavenging activity? J. Mol. Model., 2013, 19, 2593–603. 10.1007/s00894-013-1800-5 23479282

[6] Marković Z.S., Marković J.M.D., Doličanin Ć.B. Mechanistic pathways for the reaction of quercetin with hydroperoxy radical. Theor. Chem. Acc., 2010, 127, 69–80.

[7] Marković Z., Amić D., Milenković D., Dimitrić-Marković J.M., Marković S. Examination of the chemical behavior of the quercetin radical cation towards some bases. Phys. Chem. Chem. Phys., 2013, 15, 7370–7378. 10.1039/c3cp44605k 23579253

[8] Trouillas P., Marsal P., Siri D., Lazzaroni R., Duroux J.-L. A DFT study of the reactivity of OH groups in quercetin and taxifolin antioxidants: The specificity of the 3-OH site. Food Chem., 2006, 97, 679–688.

[9] Musialik M., Kuzmicz R., Pawlowski T.S., Litwinienko G. Acidity of hydroxyl groups: an overlooked influence on antiradical properties of flavonoids. J. Org. Chem., 2009, 74, 2699–2709. 10.1021/jo802716v 19275193

[10] Yang J.-G., Liu B.-G., Liang G.-Zh., Ning Zh.-X. Structure-activity relationship of flavonoids active against hard oiloxidation based on quantum chemical analysis. Molecules, 2009, 14, 46–52. 10.3390/molecules14010046 PMC6253939 19104485

[11] Galano A., Mazzone G., Alvarez-Diduk R., Marino T., Alvarez-Idaboy J. R., Russo N. Food antioxidants: chemical insights at the molecular level. Annu. Rev. Food Sci. Technol., 2016, 7, 335–352. 10.1146/annurev-food-041715-033206 26772412

[12] Alvareda E., Denis P.A., Iribarne F., Paulino M. Bond dissociation energies and enthalpies of formation of flavonoids: A G4 and M06-2X investigation. Comp. Theor. Chem., 2016, 1091, 18–23.

[13] Vinnarasi S., Radhika R., Vijayakumar S., Shankar R. Structural insights into the anti-cancer activity of quercetin on G-tetrad, mixed G-tetrad, and G-quadruplex DNA using quantum chemical and molecular dynamics simulations. J. Biomol. Struct. & Dynam., 2019, 10.1080/07391102.2019.1574239 30794082

[14] Cao G., Sofic E., Prior R.L. Antioxidant and prooxidant behavior of flavonoids: structure-activity relationships. Free Radic. Biol. Med., 1997, 22, 749–760. 10.1016/s0891-5849(96)00351-6 9119242

[15] Haenen G.R., Paquay J.B., Korthouwer R.E., Bast A. Peroxynitrite scavenging by flavonoids. Biochem. Biophys. Res. Commun., 1997, 236, 591–593. 10.1006/bbrc.1997.7016 9245694

[16] Kerry N., Rice-Evans C. Inhibition of peroxynitrite-mediated oxidation of dopamine by flavonoid and phenolic antioxidants and their structural relationships. J. Neurochem., 1999, 73, 247–253. 10.1046/j.1471-4159.1999.0730247.x 10386977

[17] Sekher Pannala A., Chan T.S., O’Brien P.J., Rice-Evans C.A. Flavonoid B-ring chemistry and antioxidant activity: fast reaction kinetics. Biochem. Biophys. Res. Commun., 2001, 282, 1161–1168. 10.1006/bbrc.2001.4705 11302737

[18] Burda S., Oleszek W. Antioxidant and antiradical activities of flavonoids. J. Agric. Food Chem., 2001, 49, 2774–2779. 10.1021/jf001413m 11409965

[19] Cadenas E., Packer L. Handbook of antioxidants (2nd Ed.). New York: Marcel Dekker, 2002.

[20] Rong Y., Wang Z., Wu J., Zhao B. A theoretical study on cellular antioxidant activity of selected flavonoids. Spectrochim. Acta A Mol. Biomol. Spectrosc., 2012, 93, 235–239. 10.1016/j.saa.2012.03.008 22484257

[21] Bogdan T.V., Trygubenko S.A., Pylypchuck L.B., Potyahaylo A.L., Samijlenko S.P., Hovorun D.M. Conformational analysis of the quercetin molecule. Scientific Notes of NaUKMA, 2001, 19, 456–460.

[22] Olejniczak S., Potrzebowski M.J. Solid state NMR studies and density functional theory (DFT) calculations of conformers of quercetin. Org. Biomol. Chem., 2004, 2, 2315–2322. 10.1039/b406861k 15305212

[23] Modelli A., Pshenichnyuk S.A. Gas-phase dissociative electron attachment to flavonoids and possible similarities to their metabolic pathways. Phys. Chem. Chem. Phys., 2013, 15, 1588–1600. 10.1039/c2cp43379f 23243660

[24] Protsenko I. O., Bulavin L.A., Hovorun D.M. Investigation of structural properties of quercetin by quantum chemistry methods. WDS'10 Proceedings of Contributed Papers, 2010, Part III, 51–54.

[25] Protsenko I.O., Hovorun D.M. Conformational properties of quercetin: quantum chemistry investigation. Repts. Natl. Acad. Sci. Ukr., 2014, N3, 153–157.

[26] Brovarets’ O.O., Hovorun D.M. Conformational diversity of the quercetin molecule: A quantum-chemical view. J. Biomol. Struct. & Dynam., 2019, 10.1080/07391102.2019.1656671 31423904

[27] Brovarets’, O.O., Hovorun, D.M. Conformational mobility of the quercetin molecule caused by the rotations of the O7H, O5H and O3H hydroxyl groups: *in silico* scrupulous study. (submitted).

[28] Antonczak S. Electronic description of four flavonoids revisited by DFT method. J. Mol. Struct. (THEOCHEM). 2008, 856, 38–45.

[29] Vasilescu D., Girma R. Quantum molecular modeling of quercetin–simulation of the interaction with the free radical t‐BuOO. Int. J. Quantum Chem., 2002, 90, 888–902.

[30] Leopoldini M., Marino T., Russo N., Toscano M. Density functional computations of the energetic and spectroscopic parameters of quercetin and its radicals in the gas phase and in solvent. Theor. Chem. Acc, 2004, 111, 210–216.

[31] Brovarets’ O.O., Hovorun D.M. Conformational transitions of the quercetin molecule *via* the rotations of its rings: A comprehensive theoretical study. J. Biomol. Struct. & Dynam., 2019, 10.1080/07391102.2019.1645734 31315531

[32] Brovarets’ O.O., Hovorun D.M. A new era of the prototropic tautomerism of the quercetin molecule: A QM/QTAIM computational advances. J. Biomol. Struct. & Dynam., 2019. 10.1080/07391102.2019.1691660 31711364

[33] Brovarets’, O.O., Protsenko, I.O., Hovorun, D.M. Comprehensive analysis of the potential energy surface of the quercetin molecule. Abstracts of the conference: "Bioheterocycles 2019, XVIII International Conference on Heterocycles in Bioorganic Chemistry" (www.bioheterocycles2019.eu; Ghent, Belgium, June 17–20, 2019), P. 84.

[34] Grytsenko O.M., Pylypchuck L.B., Bogdan T.V., Trygubenko S.A., Hovorun D.M., Maksutina N.P. Keto-enol prototropic tautomerism of quercetin molecule: quantum-chemical calculations. Farmats. Zhurn., 2003, N5, 62–65.

[35] Grytsenko O.M., Degtyarev L.S., Pilipchuck L.B. Physical-chemistry properties and electronic structure of quercetin. Farmats. Zhurn., 1992, N2, 34–38.

[36] Nikitenko N.G., Shestakov A.F. H-D exchange between quercetin and solvent in the presence of Au^I^ chloride complexes with DMSO: quantum chemical modeling. Russ. Chem. Bull., Int. Ed., 2018, 67, 1794–1802.

[37] Trouillas P., Marsal P., Siri D., Lazzaroni R., Duroux J.-C. A DFT study of the reactivity of OH groups in quercetin and taxifolin antioxidants: The specificity of the 3-OH site. Food Chem., 2006, 97, 679–688.

[38] Yang Y., Zhao J., Li Y. Theoretical study of the ESIPT process for a new natural product quercetin. Sci. Repts., 2016, 6, 32152. 27574105 10.1038/srep32152 PMC5004185

[39] Antonov L. Tautomerism: introduction, history, and recent developments in experimental and theoretical methods Tautomerism: methods and theories. Weinheim: Wiley-VCH; 2013.

[40] Antonov L. Tautomerism: A historical perspective Tautomerism: concepts and applications in science and technology. Weinheim: WILEY-VCH, 2016.

[41] Markova N., Enchev V. Tautomerism of inosine in water: is it possible? J. Phys. Chem. B, 2019, 123, 3, 622–630. 10.1021/acs.jpcb.8b11316 30604973

[42] Katritzky A.R., Hall C.D., El-Gendy B.E.M., Draghici B. Tautomerism in drug discovery. J. Comput. Aided Mol. Des., 2010, 24, 475–484. 10.1007/s10822-010-9359-z 20490619

[43] Martin Y.C. Let's not forget tautomers. J. Comput. Aided Mol. Des., 2009, 23, 693–704. 10.1007/s10822-009-9303-2 19842045 PMC2776169

[44] Taylor P.J., der Zwan G.V., Antonov L. 1st Chapter: Tautomerism: introduction, history, and recent developments in experimental and theoretical methods In book: Tautomerism: methods and theories edition. Publisher: Wiley-VCH, 2014 Editors: Antonov L., 10.1002/9783527658824.ch1

[45] Bax B., Chung C.W., Edge C. Getting the chemistry right: protonation, tautomers and the importance of H atoms in biological chemistry. Acta Crystallogr. Section D Struct. Biol., 2017, D73, 131–140. 28177309 10.1107/S2059798316020283 PMC5297916

[46] Tolosa S., Sánchez J.P., Sansón J.A., Hidalgo A. Steered molecular dynamic simulations of the tautomeric equilibria in solution of DNA bases. J. Mol. Liq., 2017, 237, 81–88.

[47] Tolosa S., Sansón J.A., Hidalgo A. Mechanisms for guanine–cytosine tautomeric equilibrium in solution *via* steered molecular dynamic simulations. J. Mol. Liq., 2018, 251, 308–316.

[48] Florián J., Leszczyński J. Spontaneous DNA mutations induced by proton transfer in the guanine∙cytosine base pairs: an energetic perspective. J. Am. Chem. Soc., 1996, 118, 3010−3017.

[49] Florián J., Hrouda V., Hobza P. Proton transfer in the adenine−thymine base pair. J. Am. Chem. Soc., 1994, 116, 1457− 1460.

[50] Florián J., Leszczyński J. Spontaneous DNA mutations induced by proton transfer in the guanine cytosine base pairs: an energetic perspective. J. Am. Chem. Soc., 1996, 118, 3010−3017.

[51] Brovarets’ O.O., Hovorun D.M. Can tautomerisation of the A∙T Watson-Crick base pair *via* double proton transfer provoke point mutations during DNA replication? A comprehensive QM and QTAIM analysis. J. Biomol. Struct. & Dynam., 2014, 32, 127–154. 10.1080/07391102.2012.755795 23383960

[52] Brovarets’ O.O., Hovorun D.M. Why the tautomerization of the G·C Watson–Crick base pair *via* the DPT does not cause point mutations during DNA replication? QM and QTAIM comprehensive analysis. J. Biomol. Struct. & Dynam., 2014, 32, 1474–1499. 10.1080/07391102.2013.822829 23909623

[53] Brovarets' O.O., Hovorun D.M. Atomistic understanding of the C·T mismatched DNA base pair tautomerization *via* the DPT: QM and QTAIM computational approaches. J. Comput. Chem., 2013, 34, 2577–2590. 10.1002/jcc.23412 23955922

[54] Brovarets' O.O., Zhurakivsky R.O., Hovorun D.M. Is the DPT tautomerisation of the long A·G Watson-Crick DNA base mispair a source of the adenine and guanine mutagenic tautomers? A QM and QTAIM response to the biologically important question. J. Comput. Chem., 2014, 35, 451–466. 10.1002/jcc.23515 24382756

[55] Brovarets' O.O., Hovorun D.M. Atomistic mechanisms of the double proton transfer in the H-bonded nucleobase pairs: QM/QTAIM computational lessons. J. Biomol. Struct. & Dynam., 2019, 37, 1880–1907. 10.1080/07391102.2018.1467795 29676661

[56] Brovarets’ O. O., Hovorun D. M. Renaissance of the tautomeric hypothesis of the spontaneous point mutations in DNA: new ideas and computational approaches In: Mitochondrial DNA—New Insights, 2018 (Vol. i, p. 13). InTechOpen: London, 2018. 10.5772/intechopen.77366

[57] Brovarets’ O.O., Hovorun D.M. Prototropic tautomerism and basic molecular principles of hypoxanthine mutagenicity: An exhaustive quantum-chemical analysis. J. Biomol. Struct. & Dynam., 2013, 31, 913–936. 10.1080/07391102.2012.715041 22962845

[58] Brovarets' O.O., Zhurakivsky R.O., Hovorun D.M. A QM/QTAIM microstructural analysis of the tautomerisation *via* the DPT of the hypoxanthine·adenine nucleobase pair. Mol. Phys., 2014, 112, 2005–2016.

[59] Masoodi H.R., Bagheri S., Ghaderi Z. The influence of Cu^+^ binding to hypoxanthine on stabilization of mismatches involving hypoxanthine and DNA bases: a DFT study. J. Biomol. Struct. & Dynam., 2019, 37, 1923–1934. 10.1080/07391102.2018.1475256 29757083

[60] Brovarets’ O.O., Hovorun D.M. Novel physico-chemical mechanism of the mutagenic action of 5-bromouracil. Ukr. Bioorg. Acta, 2009, 2, 19–23.

[61] Brovarets’ O.O., Hovorun D.M. Key microstructural mechanisms of the 2-aminopurine mutagenicity: Results of extensive quantum-chemical research. J. Biomol. Struct. & Dynam., 2019, 37, 2716–2732. 10.1080/07391102.2018.1495577 30047849

[62] Brovarets’ O.O., Pérez-Sánchez H.E., Hovorun D.M. Structural grounds for the 2-aminopurine mutagenicity: A novel insight into the old problem of the replication errors. RSC Adv., 2016, 6, 99546–99557.

[63] Brovarets’ O.O., Voiteshenko I.S., Hovorun D.M. Physico-chemical profiles of the wobble↔Watson-Crick G*·2AP(w)↔G·2AP(WC) and A·2AP(w)↔A*·2AP(WC) tautomerisations: A QM/QTAIM comprehensive survey. Phys. Chem. Chem. Phys., 2018, 20, 623–636. 10.1039/c7cp05139e 29227488

[64] Srivastava R. Theoretical studies on the electronic and optoelectronic properties of [A.2AP(w)/A*.2AP(WC)/C.2AP(w)/C*.2AP(WC)/C.A(w)/C*.A(WC)]–Au8 mismatch nucleobase complexes. Mol. Phys., 2018, 116, 263–272.

[65] Brovarets’, O.O., Protsenko, I.O., Zaychenko, G. Computational modeling of the tautomeric interconversions of the quercetin molecule. Abstracts of the International Symposium “EFMC-ACSMEDI Medicinal Chemistry Frontiers 2019” (MedChemFrontiers 2019; www.medchemfrontiers.org; June 10–13, 2019; Krakow, Poland), P. 114.

[66] Peng C., Ayala P.Y., Schlegel H.B., Frisch M.J. Using redundant internal coordinates to optimize equilibrium geometries and transition states. J. Comput. Chem., 1996, 17, 49–56.

[67] Tirado-Rives J., Jorgensen W.L. Performance of B3LYP Density Functional Methods for a large set of organic molecules. J. Chem. Theory Comput., 2008, 4, 297–306. 10.1021/ct700248k 26620661

[68] Parr R.G., Yang W. Density-functional theory of atoms and molecules. Oxford: Oxford University Press; 1989.

[69] Lee C., Yang W., Parr R.G. Development of the Colle-Salvetti correlation-energy formula into a functional of the electron density. Phys. Rev. B., 1988, 37, 785–789. 10.1103/physrevb.37.785 9944570

[70] Frisch M.J., Trucks G.W., Schlegel H.B., Scuseria G.E., Robb M.A., Cheeseman J.R., et al GAUSSIAN 09 (Revision B.01). Wallingford CT: Gaussian Inc; 2010.

[71] Brovarets’ O.O., Hovorun D.M. Atomistic understanding of the C·T mismatched DNA base pair tautomerization *via* the DPT: QM and QTAIM computational approaches. J. Comput. Chem., 2013, 34, 2577–2590. 10.1002/jcc.23412 23955922

[72] Brovarets' O.O., Zhurakivsky R.O., Hovorun D.M. Is the DPT tautomerisation of the long A·G Watson-Crick DNA base mispair a source of the adenine and guanine mutagenic tautomers? A QM and QTAIM response to the biologically important question. J. Comput. Chem., 2014, 35, 451–466. 10.1002/jcc.23515 24382756

[73] Brovarets’ O.O., Tsiupa K.S., Dinets A., Hovorun D.M. Unexpected routes of the mutagenic tautomerization of the T nucleobase in the classical A·T DNA base pairs: A QM/QTAIM comprehensive view. Front. Chem., 2018, 6, 532; 10.3389/fchem.2018.00532 30538979 PMC6277528

[74] Brovarets’ O.O., Tsiupa K.S., Hovorun D.M. Non-dissociative structural transitions of the Watson-Crick and reverse Watson-Crick А∙Т DNA base pairs into the Hoogsteen and reverse Hoogsteen forms. Sci. Repts., 2018, 8, 10371. 10.1038/s41598-018-28636-y PMC6039495 29991693

[75] Brovarets’ O.O., Tsiupa K.S., Hovorun D.M. Novel pathway for mutagenic tautomerization of classical А∙Т DNA base pairs *via* sequential proton transfer through quasi-orthogonal transition states: A QM/QTAIM investigation. PLoS ONE, 2018, 13, e0199044 10.1371/journal.pone.0199044 PMC6021055 29949602

[76] Brovarets’ O.O., Tsiupa K.S., Hovorun D.M. Surprising conformers of the biologically important A∙T DNA base pairs: QM/QTAIM proofs. Front. Chem., 2018, 6:8; 10.3389/fchem.2018.00006 29536003 PMC5835050

[77] Brovarets’ O.O., Tsiupa K.S., Hovorun D.M. The A∙T(rWC)/A∙T(H)/A∙T(rH) ↔ A∙T*(rwWC)/A∙T*(wH)/A∙T*(rwH) mutagenic tautomerization *via* sequential proton transfer: a QM/QTAIM study. RSC Adv., 2018, 8, 13433–13445. 10.1039/c8ra01446a PMC9079753 35542561

[78] Brovarets’ O.O., Tsiupa K.S., Hovorun D.M. Unexpected A∙T(WC)↔A∙T(rWC)/A∙T(rH) and A∙T(H)↔A∙T(rH)/A∙T(rWC) conformational transitions between the classical A∙T DNA base pairs: A QM/QTAIM comprehensive study. Int. J. Quantum. Chem., 2018, 118, e25674.

[79] Palafox M.A. Molecular structure differences between the antiviral nucleoside analogue 5-iodo-2`-deoxyuridine and the natural nucleoside 2`-deoxythymidine using MP2 and DFT methods: conformational analysis, crystal simulations, DNA pairs and possible behavior. J. Biomol. Struct. & Dynam., 2014, 32, 831–851. 10.1080/07391102.2013.789402 23731482

[80] El-Sayed A.A., Tamara Molina A., Alvarez-Ros M.C., Palafox M.A. Conformational analysis of the anti-HIV Nikavir prodrug: comparisons with AZT and thymidine, and establishment of structure-activity relationships/tendencies in other 6´-derivatives. J. Biomol. Struct. & Dynam. 2015, 33, 723–748. 10.1080/07391102.2014.909743 24762127

[81] Frisch M.J., Head-Gordon M., Pople J.A. Semi-direct algorithms for the MP2 energy and gradient. Chem. Phys. Lett., 1990, 166, 281–289.

[82] Hariharan P.C., People J.A. The influence of polarization functions on molecular orbital hydrogenation energies. Theor. Chim. Acta., 1973, 28, 213−222.

[83] Krishnan R., Binkley J.S., Seeger R., People J.A. Self‐consistent molecular orbital methods. XX. A basis set for correlated wave functions. J. Chem. Phys., 1980, 72, 650−654.

[84] Hratchian H.P., Schlegel H.B. Finding minima, transition states, and following reaction pathways on *ab initio* potential energy surfaces In Theory and Applications of Computational Chemistry: The First 40 Years; Dykstra C.E., Frenking G., Kim K.S., Scuseria G., Eds.; Elsevier: Amsterdam, 2005; pp 195–249.

[85] Atkins P.W. Physical chemistry. Oxford: Oxford University Press, 1998.

[86] Wigner E. Über das Überschreiten von Potentialschwellen bei chemischen Reaktionen [Crossing of potential thresholds in chemical reactions]. Zeits. Physik. Chem., 1932, B19, 203−216.

[87] Brovarets’ O.O., Hovorun D.M. Atomistic nature of the DPT tautomerisation of the biologically important C·C* DNA base mispair containing amino and imino tautomers of the cytosine: A QM and QTAIM approach. Phys. Chem. Chem. Phys., 2013, 15, 20091–20104. 10.1039/c3cp52644e 24154739

[88] Brovarets’ O.O., Hovorun D.M. DPT tautomerisation of the G·Asyn and A*·G*syn DNA mismatches: A QM/QTAIM combined atomistic investigation. Phys. Chem. Chem. Phys., 2014, 16, 9074–9085. 10.1039/c4cp00488d 24695821

[89] Keith, T.A. AIMAll (Version 10.07.01); 2010. Retrieved from aim.tkgristmill.com.

[90] Matta C.F., Hernández-Trujillo J. Bonding in polycyclic aromatic hydrocarbons in terms of the electron density and of electron delocalization. J. Phys. Chem A, 2003, 107, 7496–7504.

[91] Matta C.F., Castillo N., Boyd R.J. Atomic contributions to bond dissociation energies in aliphatic hydrocarbons. J. Chem. Phys., 2006, 125, 204103 10.1063/1.2378720 17144686

[92] Matta C.F. Modeling biophysical and biological properties from the characteristics of the molecular electron density, electron localization and delocalization matrices, and the electrostatic potential. J. Comput. Chem., 2014, 35, 1165–1198. 10.1002/jcc.23608 24777743 PMC4368384

[93] Bader R.F.W. Atoms in molecules: A quantum theory. Oxfor0064: Oxford University Press; 1990.

[94] Espinosa E., Molins E., Lecomte C. Hydrogen bond strengths revealed by topological analyses of experimentally observed electron densities. Chem. Phys. Lett., 1998, 285, 170–173.

[95] Mata I., Alkorta I., Espinosa E., Molins E. Relationships between interaction energy, intermolecular distance and electron density properties in hydrogen bonded complexes under external electric fields. Chem. Phys. Lett., 2011, 507, 185–189.

[96] Brovarets’ O.O., Yurenko Y.P., Hovorun D.M. Intermolecular CН⋯O/N Н-bonds in the biologically important pairs of natural nucleobases: A thorough quantum-chemical study. J. Biomol. Struct. & Dynam., 2014, 32, 993–1022. 10.1080/07391102.2013.799439 23730732

[97] Brovarets’ O.O., Yurenko Y.P., Hovorun D.M. The significant role of the intermolecular CH⋯O/N hydrogen bonds in governing the biologically important pairs of the DNA and RNA modified bases: a comprehensive theoretical investigation. J. Biomol. Struct. & Dynam., 2015, 33, 1624–1652. 10.1080/07391102.2014.968623 25350312

[98] Afonin A.A., Vashchenko A.V. Benchmark calculations of intramolecular hydrogen bond energy based on molecular tailoring and function-based approaches: Developing hybrid approach. Int. J. Quantum Chem., 119, 2019, e26001.

[99] Afonin A.A., Pavlov D.M., Vashchenko A.V. Case study of 2-vinyloxypyridine: Quantitative assessment of the intramolecular CeH/N hydrogen bond energy and its contribution to the one-bond 13Ce1 H coupling constant. J. Mol. Struct., 2019, 1176, 73–85.

[100] Nikolaienko T.Y., Bulavin L.A., Hovorun D.M. Bridging QTAIM with vibrational spectroscopy: The energy of intramolecular hydrogen bonds in DNA-related biomolecules. Phys Chem. Chem. Phys., 2012, 14, 7441–7447. 10.1039/c2cp40176b 22514024

[101] García-Moreno B.E., Dwyer J.J., Gittis A.G., Lattman E.E., Spencer D.S., Stites W.E. Experimental measurement of the effective dielectric in the hydrophobic core of a protein. Biophys. Chem., 1997, 64, 211–224. 10.1016/s0301-4622(96)02238-7 9127946

[102] Bayley S.T. The Dielectric Properties of Various Solid Crystalline Proteins, Amino Acids and Peptides. Trans. Faraday Soc., 1951, 47, 509–517.

[103] Dewar M.J.S., Storch D.M. Alternative view of enzyme reactions. Proc. Natl. Acad. Sci. U. S. A., 1985, 82, 2225−2229. 10.1073/pnas.82.8.2225 3857576 PMC397529

[104] Mertz E.L., Krishtalik L.I. Low dielectric response in enzyme active site. Proc. Natl. Acad. Sci. U. S. A., 2000, 97, 2081–2086. 10.1073/pnas.050316997 10681440 PMC15757

[105] Petrushka J., Sowers L.C., Goodman M. Comparison of nucleotide interactions in water, proteins, and vacuum: model for DNA polymerase fidelity. Proc. Natl. Acad. Sci. U. S. A., 1986, 83, 1559–1562. 10.1073/pnas.83.6.1559 3456600 PMC323122

[106] Samoilova A.N., Minenko S.S., Sushynskyi O.Ye., Lisetski L.N., Lebovka N.I. Optical and calorimetric studies of quercetin-doped liquid crystals: Effects of molecular aggregation. J. Mol. Liq., 2019, 10.1016/j.molliq.2018.11.050

[107] Valters R.E. Ring-chain tautomerism. Plenum Press: New York, 1985.

[108] Sigalov M.V. Ring-chain tautomerism with participation of pyridine nitrogen: The intramolecular cyclization of 2-pyridinecarboxaldehyde–indandione adducts in acidic medium. J. Mol. Struct., 2014, 1074, 302–309.

